# Supplementary material for: A spatiotemporal style transfer algorithm for dynamic visual stimulus generation
Source: Nat Comput Sci. 2024 Dec 20;5(2):155–69. doi: 10.1038/s43588-024-00746-w (PMC11860245; doi:10.1038/s43588-024-00746-w)
Supplement: Supplementary file 1 — Supplementary Table 1 and Figs. 1–3. [file 43588_2024_746_MOESM1_ESM.pdf]

nature computational science

Supplementary information https://doi.org/10.1038/s43588-024-00746-w

A spatiotemporal style transfer algorithm for dynamic visual stimulus generation

In the format provided by the authors and unedited

Supplementary Information

Label	Filename
applying cream	-mouPHwsYD8_000089_000099.mp4
balloon blowing	2loWBp9BRi0_000007_000017.mp4
blowing glass	-3ZP0DrCnOQ_000136_000146.mp4
bobsledding	--gEBElruic_000067_000077.mp4
braiding hair	-154370L9wE_000079_000089.mp4
brushing hair	-0CNBzthkZ4_000004_000014.mp4
bungee jumping	-3GJ6WdOrOw_000044_000054.mp4
catching or throwing baseball	-C_s9oUnFek_000003_000013.mp4
chopping wood	2kpaZvmqiYA_000005_000015.mp4
cleaning floor	-8JbMlvZOxs_000030_000040.mp4
cooking on campfire	-3q1QUUKZ0c_000038_000048.mp4
cracking neck	-FSQTAHxmpg_000000_000010.mp4
doing laundry	-6I88CkC6iI_000047_000057.mp4
drinking	06A7IVDdaXo_000091_000101.mp4
driving tractor	--9s8lCov-I_000002_000012.mp4
eating chips	-7784_WqE1E_000016_000026.mp4
eating spaghetti	-AEUGxV9mpg_000033_000043.mp4
exercising with an exercise ball	-9V1qe3Lk2M_000001_000011.mp4
feeding fish	-15UpGVyV4w_000001_000011.mp4
fixing hair	-3cPJnxtl7U_000095_000105.mp4
garbage collecting	02hk_9H_5hQ_000105_000115.mp4
grinding meat	-hFa8jTSaJw_000133_000143.mp4
high kick	-0lfUaTIAEs_000004_000014.mp4
hurdling	-zA6BKg9ni4_000001_000011.mp4
ice skating	11KCs9EekNo_000001_000011.mp4
kissing	-HaSHk661h4_000188_000198.mp4
marching	-0IErS_cisg_000017_000027.mp4
moving furniture	-D6-UmndVJk_000001_000011.mp4
opening present	-1Gh2yyYY7M_000019_000029.mp4
passing American football (in game)	-b6gqj_mZOs_000009_000019.mp4
petting animal (not cat)	-Aw1GOyNtlI_000013_000023.mp4
plastering	-Cf0liD1olk_000082_000092.mp4
playing basketball	0zTKKv_g0CY_000223_000233.mp4
playing chess	-AiRXJWGXZQ_000055_000065.mp4
playing cymbals	--Y25nDn2Wk_000060_000070.mp4
playing keyboard	-9gU1fIB6TM_000023_000033.mp4
playing paintball	-0ML-FXomBw_000001_000011.mp4
playing saxophone	--7VUM9MKg4_000136_000146.mp4
riding camel	-5fqwdtpSOY_000013_000023.mp4
riding mule	-9Rcf4Fzed8_000002_000012.mp4
roller skating	-1xDshoKGiw_000168_000178.mp4
sanding floor	-orecnYvpNw_000045_000055.mp4
shooting basketball	0MG3pzym-G8_000002_000012.mp4
shredding paper	-FsB_A4A1Ks_000015_000025.mp4
skiing (not slalom or crosscountry)	-fs4VkPaYRw_000050_000060.mp4
skydiving	3_e1OQlG3Qc_000052_000062.mp4
sniffing	-1fippPF1oI_000002_000012.mp4
snowmobiling	1a_Zi7bW3g8_000006_000016.mp4
spraying	-94oNUNdpQs_000012_000022.mp4
stomping grapes	-SHNC6Z1B_g_000185_000195.mp4
surfing crowd	-9bRguDPMXI_000072_000082.mp4
swimming breast stroke	0hVtX45KhS8_000075_000085.mp4
trapezing	-RpAe8io4mU_000056_000066.mp4
tying bow tie	-u1Cj9edMUI_000065_000075.mp4
washing feet	-cTzRqHS9yo_000040_000050.mp4
water sliding	-66lIjamXMA_000034_000044.mp4
archery	-UJgyiWe500_000029_000039.mp4
bandaging	3nTMc2nK_W8_000000_000010.mp4
bee keeping	0M1SkaJJcV0_000191_000201.mp4
blowing leaves	-MV_iN5WXuw_000002_000012.mp4
breading or breadcrumbing	-_3E3GBXAUc_000010_000020.mp4
busking	09ZMT8HT0DA_000030_000040.mp4

changing wheel	-sw4w8ht36s_000386_000396.mp4
cooking sausages	-LhTjRSC7Rk_000020_000030.mp4
crawling baby	-N5C2S2KW80_000018_000028.mp4
drinking beer	-A7PrCTRvOY_000263_000273.mp4
drop kicking	0qWpaHLpwpU_000003_000013.mp4
eating burger	-FbSzomWtWw_000014_000024.mp4
feeding goats	0mF342HNPTE_000034_000044.mp4
flipping pancake	-yv8c2CDbR8_000004_000014.mp4
grooming dog	-9BGMU4WYMg_000218_000228.mp4
headbanging	0QpBzgw3Nd0_000000_000010.mp4
hitting baseball	0Fet8TyoNR4_000007_000017.mp4
hurling (sport)	04LMpZuQmEM_000023_000033.mp4
ironing	3yMHI5j-BFE_000104_000114.mp4
kitesurfing	1lyLAsZLxKQ_000027_000037.mp4
laying bricks	-sf01vs2YMg_000146_000156.mp4
mowing lawn	1dhlHTnoWBw_000028_000038.mp4
passing American football (not in game)	3p7soEPNfac_000002_000012.mp4
petting cat	2eigAnTVfTU_000042_000052.mp4
playing accordion	1gQkxY9-sL4_000006_000016.mp4
playing harmonica	075wmEyfcUw_000025_000035.mp4
playing ukulele	11mwp0SbeWI_000053_000063.mp4
pumping gas	-LsxVKO78Fg_000093_000103.mp4
pushing car	-J5UQsKRvJw_000055_000065.mp4
reading newspaper	0LH6n81tyds_000010_000020.mp4
riding elephant	-5JKWL9AKOY_000004_000014.mp4
robot dancing	4ik1sNDoQcM_000029_000039.mp4
scrambling eggs	-5EKnCsR9A8_000144_000154.mp4
shaving legs	0XQ5DKQlT94_000045_000055.mp4
shooting goal (soccer)	-Ue7X0BbZWY_000215_000225.mp4
situp	-iDQZbi59iY_000009_000019.mp4
smoking hookah	1hOTjErQDjI_000002_000012.mp4
snorkeling	-4cgAtLBvTc_000003_000013.mp4
swimming butterfly stroke	-5yyKPocddE_000004_000014.mp4
tying knot (not on a tie)	-b-YkpzFphk_000111_000121.mp4
waiting in line	-5FU8vEKtyE_000009_000019.mp4
washing hair	-3uOmg8TxNI_000014_000024.mp4
watering plants	27zZJ1gOfNc_000009_000019.mp4
windsurfing	2elHqLIPUNA_000022_000032.mp4

Supplementary Table 1 Video identity used for validation analyses from the Kinetics400 dataset. First column indicates the action label associated to each video and second column the file name of the video.

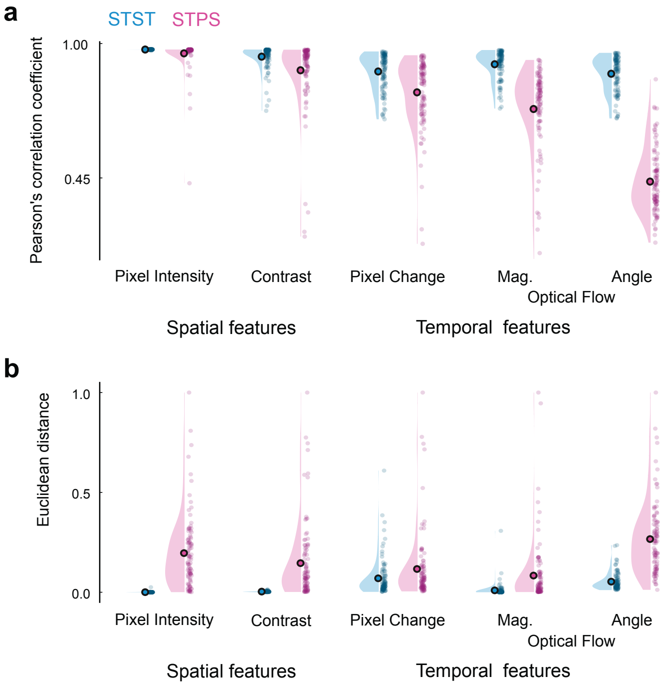

Supplementary Figure 1 I Validating low-level feature analysis on Kinetics400 dataset. Raincloud plots showing the Pearson correlation coefficient in **a** and the Euclidean distance in **b** between original videos and STST (azure) or STPS (pink) on each spatial and temporal low-level feature. Circles indicate mean values of the distribution and dots individual videos.

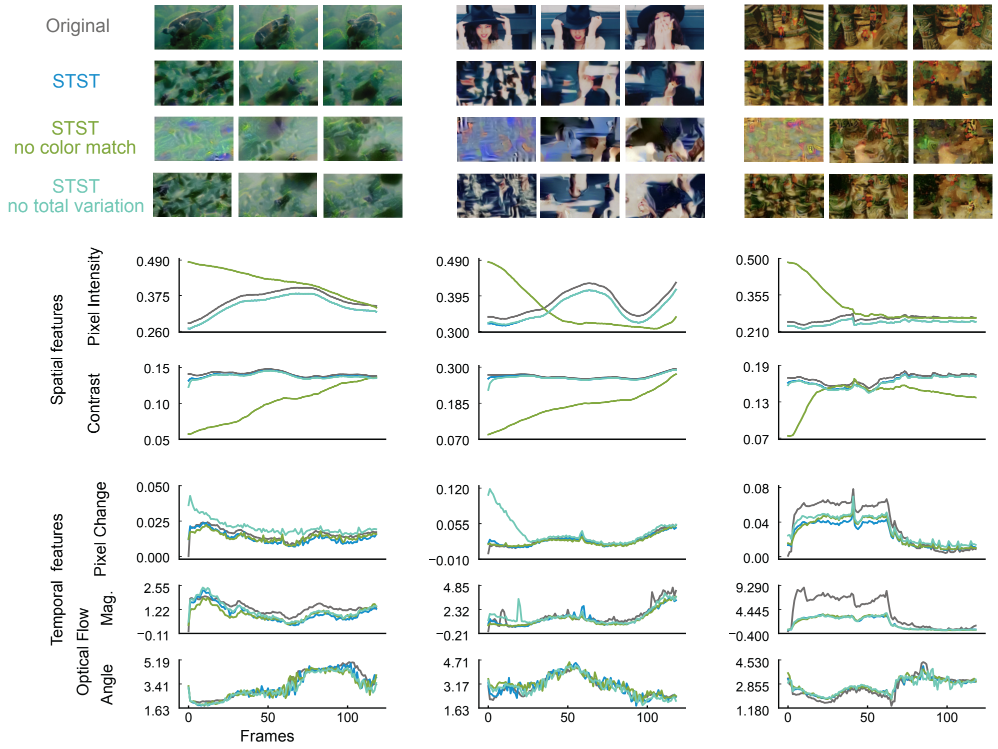

Supplementary Figure 2 I Ablation study on the color matching and total variation. Top, example frames from the original videos as well as STST with all components and its versions without color matching and without total variation. Bottom, time courses of 4 spatiotemporal features, pixel intensity and contrast as spatial low- level features and pixel change and optical flow (magnitude and angle) as temporal low-level features.

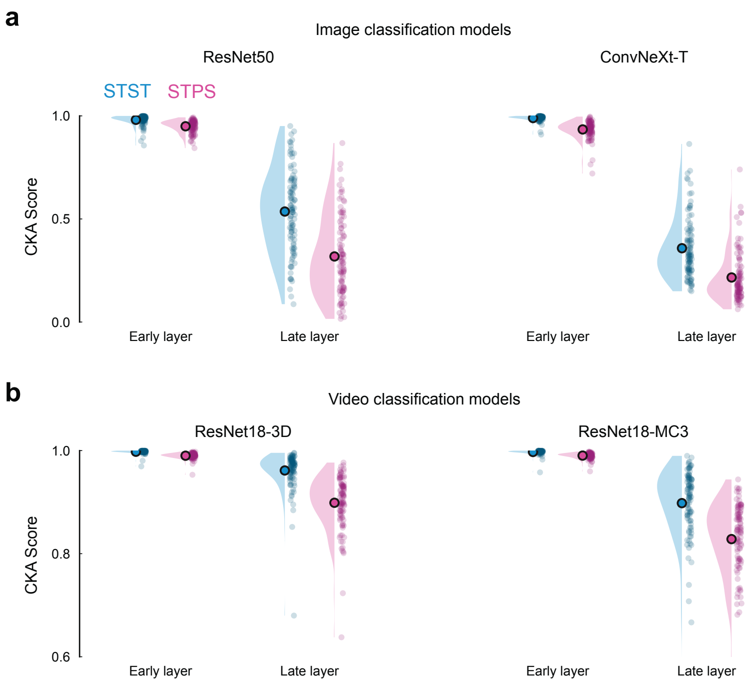

Supplementary Figure 3 | Validating metamer effect on hidden layer activations of deep visual models on Kinetics400 dataset. Raincloud plots showing the Center Kernel Alignment (CKA) score in **a** for image classification models and in **b** for video classification models between original videos and STST (azure) or STPS (pink) on early and late layer activations. Circles indicate mean values of the distribution and dots individual videos.
