# Supplementary material for: A spatiotemporal style transfer algorithm for dynamic visual stimulus generation
Source: Nat Comput Sci. 2024 Dec 20;5(2):155–69. doi: 10.1038/s43588-024-00746-w (PMC11860245; doi:10.1038/s43588-024-00746-w)
Supplement: Supplementary file 2 — Reporting Summary [file 43588_2024_746_MOESM2_ESM.pdf]

nature portfolio

Corresponding author(s): Antonino Greco, Markus Siegel

Last updated by author(s): Nov 10, 2024

Reporting Summary

Nature Portfolio wishes to improve the reproducibility of the work that we publish. This form provides structure for consistency and transparency in reporting. For further information on Nature Portfolio policies, see our Editorial Policies and the Editorial Policy Checklist.

Statistics

For all statistical analyses, confirm that the following items are present in the figure legend, table legend, main text, or Methods section.

n/a	Confirmed	
☐	☒	The exact sample size (n) for each experimental group/condition, given as a discrete number and unit of measurement
☐	☒	A statement on whether measurements were taken from distinct samples or whether the same sample was measured repeatedly
☐	☒	The statistical test(s) used AND whether they are one- or two-sided<br>*Only common tests should be described solely by name; describe more complex techniques in the Methods section.*
☒	☐	A description of all covariates tested
☐	☒	A description of any assumptions or corrections, such as tests of normality and adjustment for multiple comparisons
☐	☒	A full description of the statistical parameters including central tendency (e.g. means) or other basic estimates (e.g. regression coefficient) AND variation (e.g. standard deviation) or associated estimates of uncertainty (e.g. confidence intervals)
☐	☒	For null hypothesis testing, the test statistic (e.g. F, t, r) with confidence intervals, effect sizes, degrees of freedom and P value noted<br>*Give P values as exact values whenever suitable.*
☒	☐	For Bayesian analysis, information on the choice of priors and Markov chain Monte Carlo settings
☒	☐	For hierarchical and complex designs, identification of the appropriate level for tests and full reporting of outcomes
☐	☒	Estimates of effect sizes (e.g. Cohen's d, Pearson's r), indicating how they were calculated

Our web collection on statistics for biologists contains articles on many of the points above.

Software and code

Policy information about availability of computer code

Data collection	Behavioural data were collected using the Psychopy software v2024.1.5
Data analysis	Data analyses has been carried out using Python 3.9, TensorFlow 2.4 and Pytorch 2.2. We also used the Sentence Transformers library (version 3.0.1) for the analysis of the video caption data. The code for reproducing the STST algorithm is available at github.com/antoninogreco/STST

For manuscripts utilizing custom algorithms or software that are central to the research but not yet described in published literature, software must be made available to editors and reviewers. We strongly encourage code deposition in a community repository (e.g. GitHub). See the Nature Portfolio guidelines for submitting code & software for further information.

Data

Policy information about availability of data

All manuscripts must include a data availability statement. This statement should provide the following information, where applicable:

- Accession codes, unique identifiers, or web links for publicly available datasets
- A description of any restrictions on data availability
- For clinical datasets or third party data, please ensure that the statement adheres to our policy

The Kinetics400 dataset is available at github.com/cvdfoundation/kinetics-dataset. Generated video data are available as Supplementary Video 1-3 with this

manuscript. and bBehavioral data of the human experiments are available as Ssupplementary materialData 1 with this manuscript. Source data for Figures 2-6 are provided with this paper.

Human research participants

Policy information about studies involving human research participants and Sex and Gender in Research.

Reporting on sex and gender	- video captioning task, 8 males and 6 females<br>- 2AFC perceptual similarity task, 7 males and 6 females<br>- 2AFC spatiotemporal perceptual similarity task, stimulus set 1 9 males and 4 females, and stimulus set 2 6 males and 6 females
Population characteristics	- video captioning task N = 14, mean age = 30.4 (3.1 SD)<br>- 2AFC perceptual similarity task N = 13, mean age = 30.0 (2.8 SD)<br>- 2AFC spatiotemporal perceptual similarity task, stimulus set 1 N = 13, mean age = 30.3 (3.8 SD), and stimulus set 2 N = 12, mean age = 29.3 (3.7 SD)
Recruitment	Participants were recruited via mailing lists at the University of Tübingen, Germany, as well as from the local community. Some participants were familiar with neuroscience or psychology in general, but not with the specific hypotheses of the study. We do not expect a self-selection bias or other bias to significantly impact our results because the task relied on fundamental cognitive processes that do not require specific expertise.
Ethics oversight	All the experiments were conducted in accordance with the Declaration of Helsinki and approved by the ethics committee of the University of Tübingen. All subjects gave informed consent.

Note that full information on the approval of the study protocol must also be provided in the manuscript.

Field-specific reporting

Please select the one below that is the best fit for your research. If you are not sure, read the appropriate sections before making your selection.

☐ Life sciences ☒ Behavioural & social sciences ☐ Ecological, evolutionary & environmental sciences

For a reference copy of the document with all sections, see nature.com/documents/nr-reporting-summary-flat.pdf

Behavioural & social sciences study design

All studies must disclose on these points even when the disclosure is negative.

Study description	Quantitative study investigating human judgments on model metamers for object recognition and spatiotemporally factorized stimuli
Research sample	Sample size was chosen in accordance with the typical sample sizes used in similar studies within the field, ensuring consistency and comparability with established research practices. Participants were recruited via mailing lists at the University of Tübingen, Germany, as well as from the local community. Some participants were familiar with neuroscience or psychology in general, but not with the specific hypotheses of the study. For the three tasks, the sample statistics were the following:<br>- video captioning task N = 14, mean age = 30.4 (3.1 SD)<br>- 2AFC perceptual similarity task N = 13, mean age = 30.0 (2.8 SD)<br>- 2AFC spatiotemporal perceptual similarity task, stimulus set 1 N = 13, mean age = 30.3 (3.8 SD), and stimulus set 2 N = 12, mean age = 29.3 (3.7 SD)
Sampling strategy	We randomly sampled participants from the local community with the only constrain of having no history of psychiatric or neurological disorder and a normal or corrected to normal vision. Sample size was chosen in accordance with the typical sample sizes used in similar studies within the field, ensuring consistency and comparability with established research practices.
Data collection	We used Psychopy software to perform the experiments. Participants responded either with some keys on the keyboard or using all the keys for textual description. Only the researcher and the volunteer were present during the experiment and the researcher was blind to the assignment of the experimental conditions.
Timing	The data collection took place from july 2024 to august 2024
Data exclusions	No data were excluded
Non-participation	No participant dropped out
Randomization	We did not use any randomization as there were no experimental groups.

Reporting for specific materials, systems and methods

We require information from authors about some types of materials, experimental systems and methods used in many studies. Here, indicate whether each material, system or method listed is relevant to your study. If you are not sure if a list item applies to your research, read the appropriate section before selecting a response.

Materials & experimental systems

n/a	Involved in the study
☒	☐ Antibodies
☒	☐ Eukaryotic cell lines
☒	☐ Palaeontology and archaeology
☒	☐ Animals and other organisms
☒	☐ Clinical data
☒	☐ Dual use research of concern

Methods

n/a	Involved in the study
☒	☐ ChIP-seq
☒	☐ Flow cytometry
☒	☐ MRI-based neuroimaging
